# High disease activity in ankylosing spondylitis is associated with increased serum sclerostin level and decreased wingless protein-3a signaling but is not linked with greater structural damage

**DOI:** 10.1186/1471-2474-14-99

**Published:** 2013-03-19

**Authors:** Mariusz Korkosz, Jerzy Gąsowski, Piotr Leszczyński, Katarzyna Pawlak-Buś, Sławomir Jeka, Ewa Kucharska, Tomasz Grodzicki

**Affiliations:** 1 Division of Rheumatology, Department of Internal Medicine and Gerontology, Jagiellonian University Medical College, Polish Spondyloarthritis Initiative, Kraków, Poland; 2 Department of Internal Medicine and Gerontology, Jagiellonian University Medical College, Polish Spondyloarthritis Initiative, Kraków, Poland; 3 Department of Rheumatology and Rehabilitation, University of Medical Sciences, Poznań, Poland; 4 Department of Rheumatology and Connective Tissue Diseases, 2nd University Hospital, Bydgoszcz, Poland; 5 Vadimed Clinic, Kraków, Poland

**Keywords:** Ankylosing spondylitis, BASDAI, New bone formation, Sclerostin, Wnt

## Abstract

**Background:**

Clinical activity of ankylosing spondylitis (AS) predicts the natural course of the disease and the response to treatment. Several molecules are involved in new bone formation resulting in structural damage in patients with AS. However, the link between the clinical and molecular phenomena has not yet been fully established. The aim of the study was to investigate the relation between markers of bone remodeling and inflammation with clinical activity and structural damage in AS.

**Methods:**

We assessed the serum levels of sclerostin, Dickkopf-1 protein, Wingless protein-3a, bone morphogenic protein-7, matrix metalloproteinase-3, osteoprotegerin, bone alkaline phosphatase and inflammatory markers in 50 AS patients with high disease activity (BASDAI ≥ 4), 28 with low disease activity (BASDAI <4), and 23 healthy controls. Cervical and lumbar spine x-rays were performed in 46 patients to measure structural damage (mSASSS).

**Results:**

Sclerostin level was significantly greater in high disease activity patients than in controls. Wingless protein-3a and Dikkopf-1 protein levels were significantly lower in high activity group compared to low activity group and controls. Negative correlation was found between sclerostin and Dikkopf-1 protein in high activity group (R = −0.28, P = 0.048). The median mSASSS values were not different between patient groups.

**Conclusions:**

Higher disease activity in AS may not be per se associated with greater new bone formation.

## Background

Ankylosing spondylitis (AS) is a rheumatic disease characterized by chronic inflammation and new bone formation leading to bone remodeling, primarily in the axial skeleton [1]. Bone remodeling in AS is a dynamic process involving numerous molecules interconnected within the multilevel positive and negative feedback networks [2]. The link between inflammation and bone remodeling has not been fully explained. Tumour Necrosis Factor alpha (TNF), a pivotal pro-inflammatory cytokine in AS is responsible for induction of Dickkopf-1 (Dkk-1) and sclerostin [3], which in turn down-regulate bone formation by inhibition of Wingless proteins (Wnt) and bone morphogenic proteins (BMPs). The latter two are the key inducers of osteoblastogenesis and new bone formation [4,5].

Several other molecules are involved in bone remodeling in spondyloarthritis. Wnt up-regulates expression of osteoprotegerin (OPG), which is responsible for inhibition of osteoclastogenesis and thus bone resorption [6] by attenuating influence of receptor activator of nuclear factor kappa B ligand (RANKL) on mature osteoclasts and their precursors [7]. Matrix metaloproteinases-3 (MMP-3) is responsible for degradation of extracellular matrix of bone during inflammation [8] and was found to be negatively correlated with a serum marker of osteoblast activity and bone growth, bone–specific alkaline phosphatase (BALP) [9] in patients with spondyloarthritis who were treated with a TNF inhibitor [10].

It has been established that bone remodeling molecules, particularly Dkk-1 and sclerostin down-regulating bone formation, do not correlate, in AS patients, with CRP levels [11,12]. It is not known whether the disease activity as assessed with BASDAI, a clinical tool measuring disease activity based on information from the patient, is related to measured levels of markers of bone remodeling and degree of structural damage.

Therefore the present study aimed at testing bone remodeling and inflammatory markers in AS patients with high and low disease activity to check whether the disease activity measured by BASDAI influence the expression of bone remodeling molecules and degree of structural damage.

## Methods

The study population comprised 78 consecutive AS patients screened for eligibility to anti-TNF therapy and 23 healthy individuals. Patients were diagnosed with AS according to the modified New York criteria and were TNF inhibitor and synthetic disease modifying anti-rheumatic drugs (DMARDs) naïve. There were 50 AS patients with high disease activity and 28 with low disease activity. Patients were considered as having high disease activity if Bath Ankylosing Spondylitis Disease Activity Index (BASDAI) was ≥4, patient’s back pain visual analogue scale (VAS) ≥4 cm, patient’s global activity of disease was >5 cm and if they were refractory to at least two NSAIDs. All patients were using NSAIDs, on regular basis or on demand. The patients had predominantly axial disease with 21.8% having additional peripheral signs; 96.1% were HLA-B27 positive.

Serum samples for assessment of bone remodeling markers and markers of inflammation were obtained in all patients and controls. Samples were stored at – 80 < degrees > C. Serum level of Dkk-1, sclerostin, Wnt-3a, BMP-7, OPG, MMP-3, BALP, and CRP, TNF, IL-6 and IL-18 were measured by commercially available enzyme linked immunosorbent assay (ELISA) test (Dkk-1, BMP-7, MMP-3, BALP, R&D System Inc, Minneapolis, MN, USA; sclerostin, Wnt-3a, EIAab Science Co. Ltd., Wuhan, China; OPG, Biomedica, Wien, Austria). BASDAI was calculated in all patients. Forty six patients underwent x-ray study of cervical and lumbar spine to determine modified Stoke’s ankylosing spondylitis spine score (mSASSS) values. The Study was approved by the Jagiellonian University Bioethics Committee. All patients gave written informed consent to participate.

The database management and analyses were performed with SAS 9.2 (SAS Institute Inc., Cary, NC, USA). Means were compared either with Student’s t test or, in case of non-normal distributions, with Wilcoxon test as supplied in the proc npar1way procedure. The proportions were compared with the chi-square test, and the associations with the correlation analysis as implemented in the proc corr precedure of the SAS system. All P values are two-sided, and the 5% level of significance has been adopted throughout.

## Results

The mean (SD) age of 50 patients with high disease activity (84.62% men) was 37.8 (11.6) years, and was significantly higher than in low disease activity and control groups (Table 1). The average duration of the disease (SD) in patients with high activity was 9.0 (6.7) years and did not significantly differ from low activity group with 7.2 (5.0) years. In high activity group the median CRP (SEM) was 23.4 mg/l (3.87) and median clinical activity of the disease as assessed by BASDAI (SEM) was 7.17 (0.24) and were both significantly greater than in low activity group (Table 1).

**Table 1:** Clinical and laboratory characteristics of ankylosing spondylitis patients, with high and low disease activity and controls

	**High disease activity (BASDAI ≥ 4)**	**Low disease activity (BASDAI < 4)**	**Healthy controls**
	**n = 50**	**n = 28**	**n = 23**
age in years (SD)	37.8 (11.6)*^&^	32.0 (6.6)	32.3 (7.5)
disease duration in years (SD)	9.0 (6.7)	7.2 (5.0)	NA
BASDAI (SEM)	7.17 (0.24)*	2.79 (0.15)	NA
ESR mm/hr (SEM)	34.7 (3.09)*^&^	21.3 (3.74)^&^	6.3 (0.79)
CRP mg/l (SEM)	23.4 (3.87)*^&^	10.7 (2.24) ^&^	0.89 (0.16)
TNF alpha pg/ml (SEM)	2.03 (0.19) ^&^	2.56 (0.74) ^&^	1.77 (0.24)
IL-6 pg/ml (SEM)	6.01 (1.53) ^&^	4.4 (0.66) ^&^	0.80 (0.007)
IL-18 pg/ml (SEM)	446.25 (30.62)	418.0 (28.2)	394.0 (21.8)
Dkk-1 pg/ml (SEM)	818.7 (114.9)* ^&^	1692.7 (122.3) ^&^	1349.3 (82.7)
sclerostin ng/ml (SEM)	0.34 (0.09) ^&^	0.21 (0.12)	0.12 (0.09)
Wnt-3a ng/ml (SEM)	12.6 (2.46)* ^&^	26.3 (2.24) ^&^	16.9 (2.4)
BMP-7 pg/ml (SEM)	5.46 (0.92)	5.67 (0.97)	5.6 (1.09)
MMP-3 ng/ml (SEM)	11.6 (2.46)* ^&^	56.1 (36.7)	13.1 (1.28)
OPG pmol/ml (SEM)	4.8 (0.34) ^&^	4.0 (0.21)	3.9 (1.16)
BALP U/l (SEM)	76.9 (9.15)	57.7 (3.36)	53.4 (8.0)
mSASSS	8 (4-51)	8 (1-22)	NA
median (5–95 percentile)			

*p < 0,05 vs. low disease activity group.

^&^p < 0,05 vs. control group.

*AS* Ankylosing spondylitis, *BASDAI* Bath ankylosing spondylitis disease activity index; *ESR* erythrocyte sedimentation rate, *CRP* C-reactive protein, *TNF alpha* Tumour necrosis factor alpha, *IL-6* interleukin 6, *IL-18* Interleukin 18, *Dkk-1* Dickkopf-1 protein, *Wnt-3a* wingless protein-3a, *BMP-7* Bone morphogenic protein-7, *MMP-3* Matrix metalloproteinase 3, *OPG* Osteoprotegerin, *BALP* Bone alkaline phosphatase, *mSASSS* Modified Stoke’s ankylosing spondylitis spine score, *NA* Not applicable, *SD* Standard deviation, *SEM* Standard error of the mean.

The mean serum levels (SEM) of the seven studied markers of bone remodeling and four markers of inflammation in three studied groups are given in Table 1.

Briefly, sclerostin serum level was significantly higher in AS patients with high disease activity in comparison to healthy subjects. Wnt-3a and Dkk-1 level were significantly lower in high activity group compared to low activity group and healthy controls. There were no significant correlations between remodeling molecules, inflammatory markers, BASDAI and mSASSS (including lack of correlation between CRP and sclerostin) except negative correlation between sclerostin and Dkk-1 in high disease activity group (R = −0.28, P = 0.048).

Levels of CRP, TNF and IL-6 were significantly higher in each AS group than in controls, but were not different between AS groups, except CRP which was higher in active group.

To assess the interaction between disease activity and structural damage in AS patients, we checked the mean mSASSS values in groups with high (BASDAI ≥ 4) and low (BASDAI < 4) clinical activity. The median mSASSS values were not significantly different between groups (Table 1).

## Discussion

In this cross-sectional study we showed that serum level of sclerostin was significantly higher in AS patients with high disease activity than in healthy subjects. Although numerically greater, the difference between level of sclerostin in the high activity group and the low activity group did not reach statistical significance (0.34 vs. 0.21 ng/ml, P = 0.09, high vs. low AS activity, respectively). The sclerostin level in the group with low disease activity was not different from the control subjects. Higher level of sclerostin in active AS group agreed with higher inflammation activity as expressed by significantly greater CRP level in these patients in comparison to low activity group and healthy controls. However, no correlation between sclerostin and CRP was found, suggesting that sclerostin may not be directly linked to acute phase response. Likewise level of Dkk-1 was not correlated with CRP in our study, which is concordant with other published data [11,12]. This confirms that interaction between inflammatory markers and inhibitors of bone formation in AS is complex and might not translate into simple correlations.

To check whether a novel, more objective measures of disease activity would have more bearing on the results, we retrospectively substituted BASDAI with the Ankylosing Spondylitis Disease Activity Score incorporating the CRP level (ASDAS-CRP) [13]. We found no correlation between ASDAS-CRP and sclerostin level. Moreover, we found no difference in sclerostin levels when ASDAS-CRP 3.5 cut-off was used instead of BASDAI 4.0 cut-off. We also compared the mSASSS scores in these groups and we found no statistically significant difference between median values of mSASSS (P = 0.13), a finding reproducing our main results where BASDAI was used as clinical measure of AS activity.

In contrast to our results, Appel et al. reported serum level of sclerostin to be significantly lower in 46 AS patients with mean disease duration (SD) of 5.3 years (2.6) in comparison to healthy controls [14]. In a subgroup analysis level of sclerostin over 2-year follow-up was significantly higher in patients with no syndesmophyte development than in patients with new syndesmophyte growth. However, the authors of that report did not mention the BASDAI and CRP values in the studied groups which precludes direct comparison with our high activity AS group. Furthermore, patients were selected from a database on the grounds that their blood samples and x-ray data were available for 2 years in yearly intervals (0, 1 and 2 year) to capture least significant change in syndesmophyte growth, which may have introduced a bias. Therefore we assume that majority of their patients, having lower level of sclerostin than healthy controls, probably had low disease activity and would have had possibly higher mSASSS values, which were not reported again. They conclude that low sclerostin level in AS was found to be associated with syndesmophyte formation and may be useful as a biomarker to assess the risk of developing syndesmophytes in AS [14]. This contrasts our results where higher sclerostin level in high disease activity group was not associated with lower level of syndesmophyte formation since mSASSS in our both groups was comparable.

To our best knowledge, only one study addressed the issue of BASDAI influence on sclerostin and Dkk-1 serum level in AS patients [12]. In the AS groups with BASDAI ≥ 4 and BASDAI < 4, the authors reported no differences of Dkk-1 and sclerostin levels in the former contrary, the latter concordant with our results. In addition they found no differences between all AS patients and healthy controls with regard to sclerostin and Dkk-1 serum concentration. However 23 out of 55 patients studied were already receiving TNF inhibitor treatment, for a median of 24 (16–33) months, which may have influenced the results, in particular CRP and BASDAI. In addition, the radiographic data were not reported, making direct comparisons with our data difficult.

It has been shown in an animal model of chronic inflammatory arthritis that TNF induces skeletal expression of Dkk-1 which in turn triggers sclerostin production [3], both molecules being potent inhibitors of new bone formation. Interestingly, in our study both the serum level of TNF and sclerostin were not statistically different across the high and low AS activity group. This might suggest that there are other molecular mechanisms, than related to acute-phase response, responsible for modulation of Dkk-1 and sclerostin serum level in AS patients with high disease activity. Moreover, it may be hypothesized that there are high and low producers of sclerostin among AS patients which requires independent confirmation. In addition, our negative correlation between sclerostin and Dkk-1 in high disease activity group (R = −0.28, P = 0.048) is against the Heiland et al. conclusion that neutralization of Dkk-1 resulted in decreased expression of sclerostin in osteocytes [3]. The possible explanation of the difference between our data and those reported by Heiland et al. rests with the fact that we used serum and not bone tissue samples.

Wnt-3a level was significantly lower in our AS group with high disease activity compared to AS group with low disease activity and healthy controls and this is in line with formerly reported inhibitory effect of sclerostin on Wnt-3a [3]. In low disease activity group we found increased Dkk-1 serum level which may be a counter-balancing mechanism attenuating the Wnt signaling, active in AS with low activity of inflammation. This hypothesis was suggested by Daoussiss et al. who found increased level of Dkk-1 after 12 weeks of anti-TNF treatment in 8 patients with AS resulting in significantly decreased CRP level [15]. Indeed, in our data, Wnt-3a and Dkk-1 levels in low AS activity group with low CRP level were the highest and significantly greater than in high activity group and healthy controls. However, it needs to be mentioned that our low-CRP/low AS activity group cannot be directly compared with the findings in the post anti-TNF treatment patients reported by Daoussiss et al.

Our results must be viewed in the context of their limitations. The sample size was moderate, although in line with other research in this field reported in literature. In our study we chose Wnt-3a from among other Wnt molecules. However, the results obtained for this particular protein cannot be viewed as representative for the rest of the Wnt family. Such selection was based on search of the available literature and on its key role in direct bone formation, most likely due to activation of canonical Wnt signaling pathway [16,17]. The remaining issue is whether serum level of sclerostin is stable to warrant the conclusion that its elevated level influences mSASSS, the latter reflecting rather longstanding process of bone remodeling. Although we do not have longitudinal data, the study by Appel et al. provide evidence that sclerostin serum level was continuously raised for two years in a group with no radiographic progression of syndesmophytes [14].

## Conclusions

In conclusion, serum level of sclerostin was elevated in high disease activity AS group and was associated with decreased serum level of Wnt-3a. Median structural damage measured by mSASSS in both groups was comparable. This suggests that sclerostin might be responsible for suppression of molecules inducing new bone formation in AS. It also indicates that higher clinical activity in AS might not be per se associated with greater new bone formation.

## Competing interests

The authors declare that they have no competing interests.

## Authors’ contributions

MK was responsible for study design, patients’ enrollment, calculating mSASSS, analysis of investigated data, and drafting manuscript; JG was responsible for study design, statistical analysis and drafting manuscript; PL, KPB, SJ and EK were involved in patients’ enrollment and analysis of investigated data; TG was responsible for study design and critically reviewed manuscript. All authors critically read and approved the final manuscript.

## Pre-publication history

The pre-publication history for this paper can be accessed here:

http://www.biomedcentral.com/1471-2474/14/99/prepub
